# Effect of Intravenous Lipid Emulsion Therapy on Xenobiotic Plasma Partitioning and Short-Term Neurological Outcome in Companion Animals with Suspected Neurotoxicosis: A Case Series

**DOI:** 10.3390/ani16091352

**Published:** 2026-04-28

**Authors:** Arne Voorhorst, Céline Pouzot-Nevoret, Johannes C. M. Vernooij, Julie Combet-Curt, Philippe Berny, Arjen Koppen, Joris H. Robben

**Affiliations:** 1 Section of Emergency and Intensive Care Medicine, Department of Clinical Sciences, Faculty of Veterinary Medicine, Utrecht University, P.O. Box 80.154, 3508 TD Utrecht, The Netherlands; a.voorhorst@uu.nl; 2 Intensive Care Unit (SIAMU), Agressions Pulmonaires et Circulatoires dans le Sepsis, VetAgro Sup, Campus Vétérinaire de Lyon, Université de Lyon, 69280 Marcy-l’Étoile, France; 3 Section of Farm Animal Health, Department of Population Health Sciences, Faculty of Veterinary Medicine, Utrecht University, 3508 TD Utrecht, The Netherlands; j.c.m.vernooij@uu.nl; 4 Centre Hospitalier Vétérinaire Saint-Martin, 74350 Allonzier-la-Caille, France; j.combetcurt@gmail.com; 5 Interactions Cellules Environnement, VetAgro Sup, Campus Vétérinaire de Lyon, Université de Lyon, 69280 Marcy-l’Étoile, France; 6 Dutch Poisons Information Center, University Medical Center Utrecht, 3584 CX Utrecht, The Netherlands; a.koppen@umcutrecht.nl

**Keywords:** log *P*, lipid sink, lipid shuttle, neurotoxin, feline, canine

## Abstract

Intravenous lipid therapy (ILE) is used to treat neurotoxicosis in companion animals, but clinical evidence for in vivo xenobiotic partitioning and benefit remains limited. This study evaluated associations between the log n-octanol/water partition coefficient (log *P*), in vivo plasma partitioning, and early neurological outcome. In this case series from eight veterinary hospitals, dogs and cats with suspected neurotoxicosis received ILE. Blood collected before and at the end of infusion was separated into lipid and aqueous fractions and analyzed by gas chromatography–mass spectrometry. Log *P* values were retrieved from PubChem. Outcome (improvement vs. no-improvement) was the change from ILE-start to 4–6 h later. Thirty-four cases were analyzed (27 dogs and seven cats; 17 different xenobiotics were identified). At the end of infusion, xenobiotic lipid fraction concentrations exceeded aqueous concentrations in 28/34 cases, and log *P* did not correlate with the lipid-to-aqueous ratio. Improvement occurred in 14/34 animals and was associated with higher lipid-to-aqueous xenobiotic ratios (geometric mean ratio 5.7; 95% CI 1.73–19.05; *p* = 0.007). Overall, in vivo lipid sequestration was frequent and was associated with early neurological improvement, whereas log *P* alone did not predict partitioning or outcome.

## Simple Summary

In some poisoning cases of dogs and cats, veterinarians use a fat-based intravenous infusion to help remove toxins that have already been absorbed through the gut. The idea behind fat-based infusion therapy is that fat can temporarily bind fat-soluble toxins in the bloodstream, potentially reducing their effect. The literature that studies this idea in a clinical setting is limited. In this study, we looked at 34 dogs and cats treated with this fat-based solution at eight veterinary hospitals. We measured how much of the toxin was in the fat-based portion of the blood versus the water-based portion. In most cases, toxins are collected into the fat portion. We found that animals in which neurological symptoms improved within a few hours had higher fat-to-water toxin ratios. A standard measure of how fat-soluble a toxin is (the log n-octanol/water partition coefficient or log *P*) did not accurately predict whether animals would improve. This suggests that while fat can bind certain toxins, predicting which animals will benefit from this therapy may require more than just fat-solubility data.

## 1. Introduction

Acute neurotoxicosis in companion animals is a common, potentially life-threatening emergency that can require rapid therapeutic interventions [1,2,3]. Conventional decontamination and supportive care suffice in most patients, but severe intoxications often require adjunctive therapies such as intravenous lipid emulsion therapy (ILE) early in the clinical course [4,5,6,7,8]. Intravenous lipid emulsion therapy has demonstrated efficacy in local-anesthetic systemic toxicity (LAST) in both experimental animals and humans and has been incorporated into guidelines for severe LAST [9,10,11,12,13]. Use of ILE has since extended to non-LAST poisonings, particularly those causing neurotoxicosis. Studies of clinical efficacy remain heterogeneous and sometimes contradictory, complicating clinical decision-making [5,8,13,14,15,16,17,18,19,20,21,22,23,24,25]. Many different poisonings could potentially benefit from ILE therapy [4].

Understanding ILE’s mechanisms of action is essential to guide its use across different poisonings, for which several mechanisms have been proposed [19]. The “lipid sink” hypothesis posits that an intravascular lipid phase sequesters lipophilic xenobiotics from the largely aqueous plasma, reducing pharmacologically active (“aqueous” or “free”) concentrations and promoting diffusion away from target tissues such as the central nervous system [10,19]. The “lipid shuttle” hypothesis extends this concept by proposing that lipid-bound xenobiotics are subsequently continuously transported to organs for metabolism and elimination [26,27,28]. Clinical evidence directly linking xenobiotic sequestration to clinical outcome remains limited and often contradictory [6,7,8,19,29].

The log_10_ n-octanol/water partition coefficient (log *P*) is commonly used to estimate lipophilicity and predict partitioning behavior of a substance [30,31]. A relation between a higher log *P* and reduction in the xenobiotic concentration in the aqueous fraction has been demonstrated in vitro [29,32]. Several in vitro and in vivo studies, primarily on local anesthetics, have demonstrated decreased aqueous-phase concentrations or lipid-phase sequestration for high-log *P* toxins [10,27,28,30,33,34,35,36]. Whether log *P* accurately predicts in vivo lipid sequestration of xenobiotics other than local anesthetics remains uncertain [8,19,29].

The primary objective was to assess xenobiotic partitioning between lipid and aqueous plasma fractions after ILE in a clinical setting. Secondary objectives were to evaluate whether this partitioning was associated with early neurological improvement and whether an in vitro measure of lipophilicity, log *P*, predicted in vivo plasma partitioning or short-term neurological outcome.

## 2. Materials and Methods

### 2.1. Study Design

This was a prospective, multicenter case series conducted at 8 European veterinary referral hospitals (see Acknowledgements). All procedures complied with institutional animal care requirements and relevant veterinary ethics guidance.

### 2.2. Study Population

All client-owned dogs and cats with acute neurological signs that were presented to the 8 hospitals between June 2020 and November 2021 were eligible for enrolment if: there was both a history suggestive of neurotoxin exposure, witnessed or suspected, and a clinical presentation consistent with neurotoxicosis (e.g., acute tremors, ataxia, seizures, and altered mentation). Patients with pre-existing conditions that contraindicated lipid infusion, e.g., congestive heart failure, known lipid-metabolism disorders (e.g., hypertriglyceridemia), and hypersensitivity to emulsion components, were not enrolled.

### 2.3. Treatment Protocol

A 20% soybean oil emulsion (Medialipid^®^ 20% [B. Braun, Melsungen, Germany] or Intralipid^®^ 20% [Fresenius Kabi, Bad Homburg, Germany]) was administered as a 1.5 mL/kg intravenous bolus over 1 min, followed by a continuous infusion at 0.25 mL/kg/min for 60 min. Up to 2 identical additional cycles were permitted at the treating clinician’s discretion if deemed clinically indicated and if post-centrifugation plasma was not macroscopically lipemic [6,7].

ILE was administered off-label at the discretion of the attending clinician, in accordance with local regulations and institutional policies. Further care followed the attending clinician’s discretion.

### 2.4. Blood Sampling and Processing

Samples were drawn immediately before initiation of intravenous lipid emulsion (ILE) therapy (baseline, T0), immediately after the infusion ended (T1), and 4 h after completion of the infusion (T5). Depending on the patient size, 2–4 mL of blood was collected by venipuncture or via an indwelling catheter into ethylenediaminetetraacetic acid (EDTA) tubes. Whole blood was centrifuged as soon as possible; plasma was separated and stored at −20 °C, then shipped to the laboratory Interactions Cellules Environnement, VetAgro Sup, Campus Vétérinaire, Marcy-l’Étoile, France, for further analysis.

After thawing, post-ILE samples (T1 and T5) were centrifuged at 9000× *g* for approximately 10 min at room temperature to obtain an upper lipid layer and a lower aqueous plasma fraction. The upper lipid layer was carefully aspirated first. Any visible interphase was avoided and discarded. The remaining aqueous plasma fraction was then collected for analysis. To reduce cross-contamination, aspiration was performed cautiously and not up to the visible phase boundary. Aliquots of pre-ILE plasma (T0) and of the lipid and aqueous fractions from T1 and T5 were subjected to liquid–liquid extraction using ToxiVials type A and B (Advion Interchim Scientific^®^, Montluçon, France). When sample volume was insufficient, ultrapure water was added to reach the minimum extraction volume. Samples were then mixed and centrifuged at 2500× *g* for 5 min at 20 °C. The organic supernatant was collected into concentration cups, evaporated to dryness at 37 °C, and reconstituted in 0.5 mL ethanol.

### 2.5. Xenobiotic Identification and Quantification

Analyses were performed using an Agilent gas chromatography–mass spectrometry system (GC–MS 5973; Agilent Technologies Inc., Wilmington, DE, USA) equipped with a 30 m capillary column and helium carrier gas, with splitless injection (3 µL). The oven program was 75 °C (0 min), increased at 20 °C min^−1^ to 200 °C (2 min hold), then at 8 °C min^−1^ to 250 °C (3 min hold), and finally at 25 °C min^−1^ to 280 °C (15 min hold), for a total run time of 33.7 min. Data were acquired in full-scan and selected ion monitoring (SIM) modes. Tentative full-scan identifications were confirmed against authentic standards or pharmacy-grade products. Quantification in SIM mode used four diagnostic ions per analyte following Lemarchand et al. (2012) and an in-house protocol adapted for lipid matrices [37]. Routine SIM covered >30 xenobiotics, with supplementary calibrations added as needed. Routine analytical quality control was applied throughout. Blank samples were run between clinical samples. Analytical standards were included in each analytical series for most drugs, and a reference drug was used when no analytical standard was available. Spiked samples were included in each analytical series, with recoveries > 70%. Calibration curves were constructed using five concentration points and accepted at r^2^ > 0.99. No internal standards were used because the method was applied across a broad multi-analyte panel.

### 2.6. Physicochemical Data

Log *P* was retrieved from PubChem (accessed April 2024; see also Data Availability below). If multiple xenobiotics were detected in a case, the xenobiotic with the highest measured concentration at T1 was used as the primary analyte for case-level analyses to reduce the influence of low-concentration measurements near the limit of quantification with potentially more variability.

### 2.7. Clinical Outcome

Neurological examinations were performed by the attending clinicians from the pre-ILE baseline (T0) to 4–6 h after discontinuation of the ILE continuous-rate infusion. Findings were recorded in the electronic medical record at each center and transcribed onto prespecified data-collection forms. Two experienced reviewers (AV, JCC), blinded to xenobiotic identifications and all fraction-specific concentrations, independently reviewed the clinical data and assigned the short-term neurological outcome as improved or not improved. Discordant assignments were resolved by discussion to reach a consensus for analysis.

### 2.8. Data Handling and Statistical Analysis

Descriptive results are reported as median (range), unless stated otherwise. Values for xenobiotic concentrations below the level of quantification (LOQ, 0.01 mg/L) were displayed in tables as “<LOQ” and assigned 0.005 mg/L (½ LOQ) for analyses. Associations between log *P* and post-ILE xenobiotic concentrations, and between log *P* and the end-of-infusion lipid-to-aqueous ratio, were examined using Spearman’s rank correlation. General linear models were fitted to log10-transformed outcomes to examine these relationships. Model coefficients (*β*) were back-transformed so that 10^*β* (the geometric mean ratio, GMR) quantifies the multiplicative change in the outcome per +1 unit of log *P* or early neurological improvement (improved/not improved). Model validity was assessed by visual inspection of the residuals.

Association between rounded log *P* values and early neurological improvement (improved/not improved) was tested using a two-sided Fisher’s exact test.

Inter-observer agreement for binary and ordinal ratings was summarized as percent agreement with exact 95% confidence intervals (CIs) and a two-sided exact binomial test versus 50% agreement. Results are presented as estimates with 95% CIs. A *p* = 0.05 was considered significant. Given the small sample size and the very small toxin- and species-specific subgroups, no multivariable adjustment for additional covariates was performed. All analyses were exploratory and intended to describe patterns and generate hypotheses. All statistical analyses were performed in R Version 4.2.3 [38].

## 3. Results

### 3.1. Study Population

**Figure 1:**
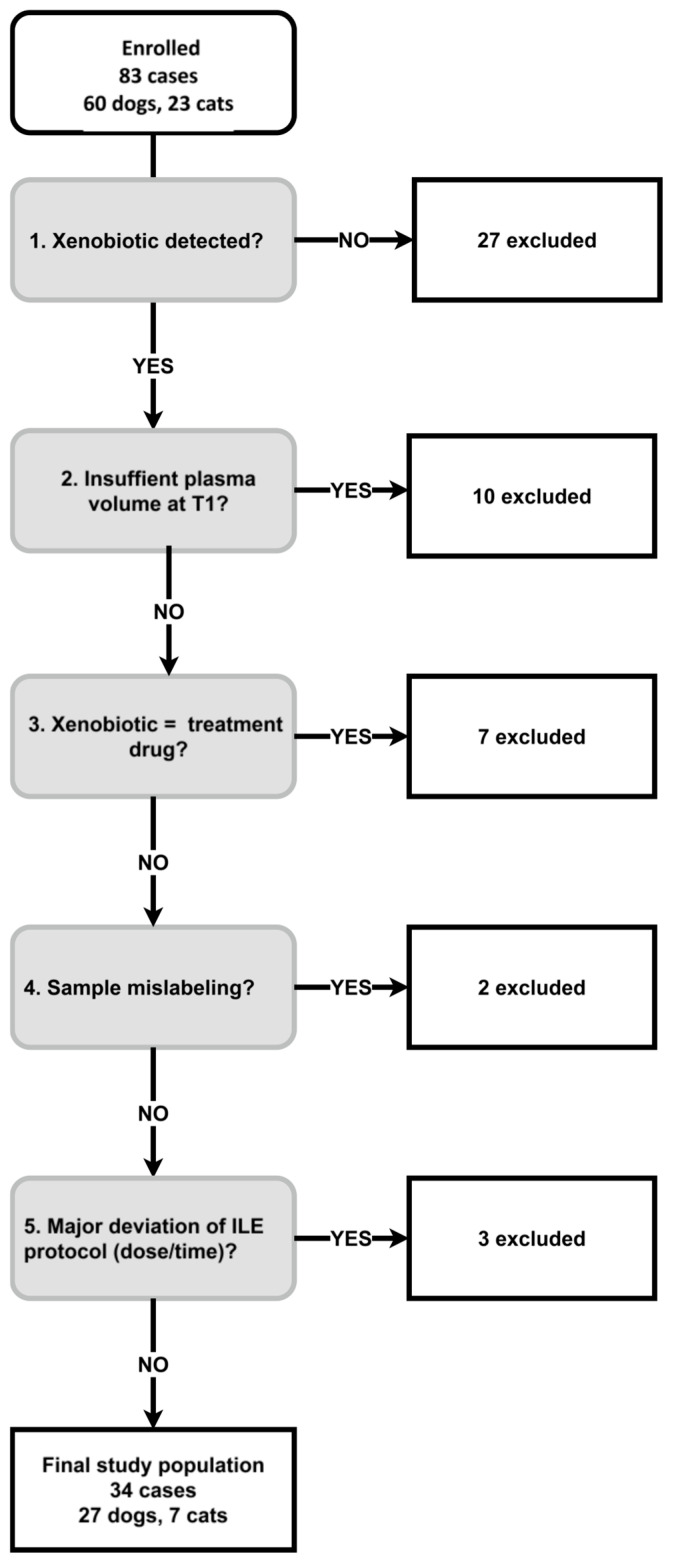
Stepwise exclusion flow chart for the final study population (27 dogs and seven cats) with suspected neurotoxicosis receiving intravenous lipid emulsion therapy. Step 3: if the identified xenobiotic was a drug that the animal was treated with, like acepromazine, it was excluded. Step 5: a total intravenous lipid emulsion dose of 15–30 mL/kg completed within 70 min (bolus plus short CRI) was sufficient for inclusion; other protocols were excluded.

Eighty-three animals were enrolled (60 dogs, 23 cats) that received a total of 85 ILE treatments with one cat undergoing three cycles. After applying post hoc exclusion criteria, 34 cases (27 dogs, seven cats) remained for further analysis (Figure 1).

For paired T0–T1 comparisons, one dog was excluded because no T0 sample was available, leaving 33 cases (26 dogs, seven cats). Given the small number of cats that remained after applying exclusion criteria, statistical analyses were limited to dogs and all animals combined.

Of the dogs, 22 of 27 were purebred and five mixed-breed. Most common breeds were Labrador Retriever (n = 4) and American Staffordshire Terrier (n = 2). Median age was 2.8 years (0.3–8.8) and body weight 15.6 (2.0–40.0) kg; 17 dogs were female (seven spayed, 10 intact) and 10 male (three neutered, seven intact). Four of seven cats were Domestic Shorthair and three had no breed recorded. Median age was 0.9 years (0.2–15.2) and body weight 4.3 (1.5–4.9) kg. Three cats were female (one spayed, two intact) and four male (one neutered, three intact).

### 3.2. Treatments

Twenty-one of 34 ILE treatments strictly adhered to the prespecified treatment protocol. Because strict adherence to the prespecified protocol was uncommon, the dosing-based inclusion criteria were broadened post hoc to include a bolus followed by a continuous-rate infusion totaling 15–30 mL/kg within 70 min.

In addition to ILE therapy, sedatives were the most commonly co-administered supportive treatments (16/34), followed by intravenous crystalloids (13/34) and anti-emetics (10/34). Other treatments than intravenous lipid emulsion are provided in Supplementary Table S2.

### 3.3. Xenobiotic Identification, Quantification and Physicochemistry

The xenobiotics identified at T1, their frequency, and their log *P* are shown in Table 1.

Seventeen different xenobiotics were identified at T1. Permethrin (9/34) and Δ^9^-THC (6/34) were the most common. Log *P* values ranged from 1 to 7.0, with a median of 4.1.

Samples from T5 were not analyzed further because the lipid phase was usually absent or too small for reliable fraction-specific quantification.

In 14/33 cases (42%), the xenobiotic concentration at either T0, the T1 aqueous or lipid phase was below the lower limit of quantification. The T0 plasma concentration exceeded the T1 aqueous concentration in 12/33 cases (36%), was lower in 11/33 (33%), and was equal in 10/33 (30%). Individual fraction concentrations at T0 and T1 are provided in Supplementary Table S2.

**Figure 2:**
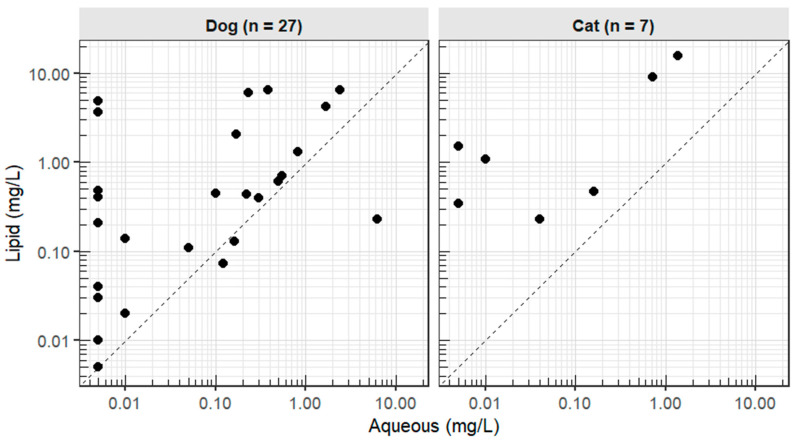
Xenobiotic plasma concentrations in lipid versus aqueous fraction following intravenous lipid emulsion therapy in 34 companion animals with suspected neurotoxicosis. The limit of quantification (LOQ) was 0.01 mg/L; values reported as “<LOQ” were plotted at 0.005 mg/L (½ LOQ).

At T1, lipid phase concentrations exceeded aqueous in 28/34 cases (82%); the median lipid-to-aqueous ratio was 5.1 (0.04–978) (Figure 2).

### 3.4. Log P and Xenobiotic Concentrations

**Figure 3:**
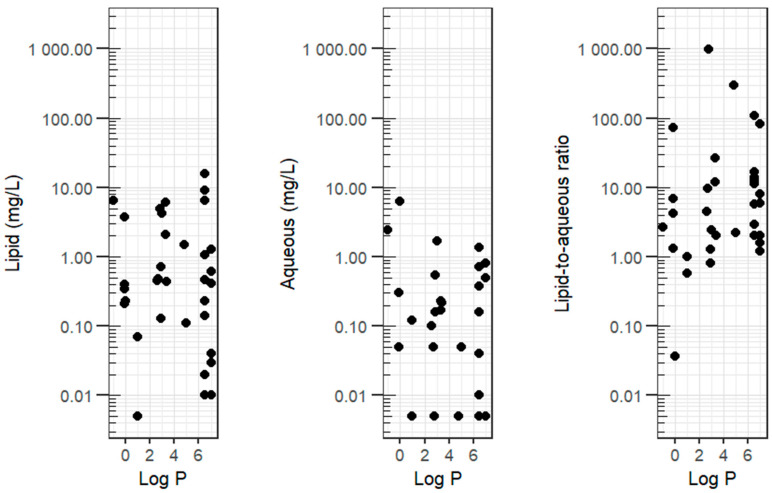
Xenobiotic plasma concentrations (lipid, aqueous, lipid-to-aqueous ratio) versus log *P* following intravenous lipid therapy in 27 dogs and 7 cats with suspected neurotoxicosis.

Scatterplots of log *P* versus T1 lipid concentration, T1 aqueous concentration, and the T1 lipid-to-aqueous ratio showed wide dispersion (Figure 3).

- Log *P* = base-10 logarithm of the n-octanol/water partition coefficient (derived from PubChem);
- Lipid-to-aqueous ratio = lipid fraction concentration divided by the aqueous fraction concentration.

Spearman’s rank correlations were weak and not statistically significant in dogs and in all animals combined (Table 2).

In linear models, each one-unit increase in log *P* in dogs was associated with a 29% lower T1 aqueous concentration (GMR 0.71; 95% CI 0.54–0.94; *p* = 0.02). This association was weak and not statistically significant when cats were included. No significant associations were identified in the linear models for T1 lipid concentration or for the T1 lipid-to-aqueous ratio in dogs or in all animals combined (Table 3).

### 3.5. Short-Term Neurological Outcome, Xenobiotic Concentrations, and Log P

**Figure 4:**
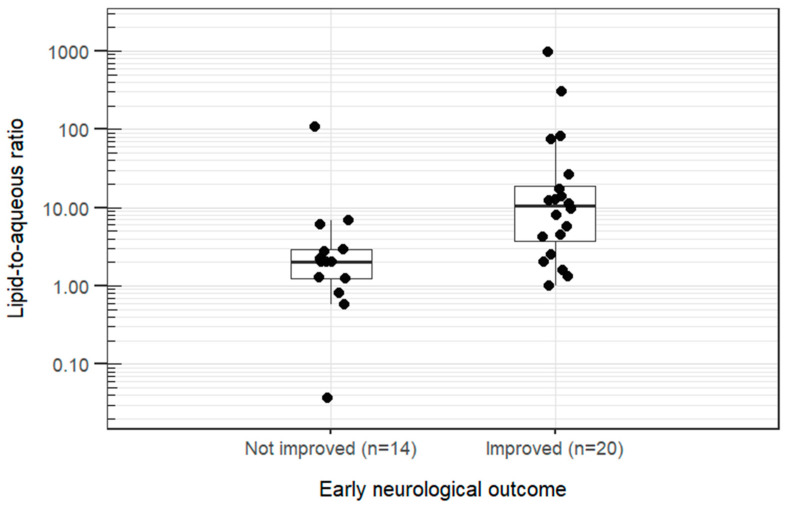
Xenobiotic lipid-to-aqueous ratio versus early neurological outcome following intravenous lipid emulsion therapy in 27 dogs and 7 cats with suspected neurotoxicosis.

The most common clinical signs at presentation were generalized tremors (21/34, 62%), ataxia (18/34, 53%), altered consciousness (15/34, 44%), localized tremors (6/34, 18%), and seizures (5/34, 15%). For early neurological clinical outcome (improved vs. not improved), inter-observer agreement was 23/34 (95% CI, 0.50–0.83; *p* = 0.058). All discrepancies were resolved satisfactorily by consensus before final analyses. Improvement was recorded in 14/34 cases. Animals that improved had higher T1 lipid-to-aqueous ratios than those that did not improve it (dogs: GMR 8.09; 95% CI 2.31–28.38; *p* = 0.003, 709% higher; dogs and cats: GMR 5.75; 95% CI 1.73–19.05; *p* = 0.007, 475% higher (Figure 4)).

- Lipid-to-aqueous ratio = lipid fraction concentration divided by the aqueous fraction concentration;
- Boxes show median and range from the first to the third quartile; whiskers extend to values within 1.5 times the interquartile range;
- Early neurological outcome was defined as the neurological improvement from the start of intravenous lipid emulsion administration to 4–6 h afterwards. The outcome was assessed independently by two observers; disagreements were resolved by consensus.

Analyses of the lipid and aqueous fractions considered separately did not identify significant between-group differences. 

**Figure 5:**
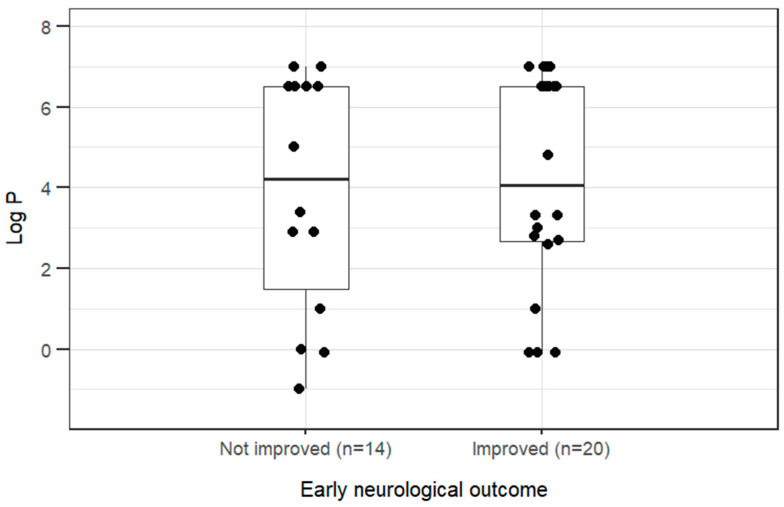
Log *P* versus early neurological outcome following intravenous lipid emulsion therapy in 27 dogs and 7 cats with suspected neurotoxicosis.

Figure 5 presents the log *P* versus early neurological outcome. No association was found between rounded log *P* values (*p* = 0.981).

- Log *P* = base-10 logarithm of the n-octanol/water partition coefficient (derived from PubChem);
- Boxes show median and range from the first to the third quartile; whiskers extend to values within 1.5 times the interquartile range;
- Early neurological outcome was defined as the neurological improvement from the start of intravenous lipid emulsion administration to 4–6 h afterwards. Outcome was assessed independently by two observers; disagreements were resolved by consensus.

## 4. Discussion

In this multicenter clinical case series, xenobiotic concentrations at the end of ILE treatment were usually higher in the plasma lipid fraction than in the aqueous fraction. In addition, a higher lipid-to-aqueous ratio was associated with early neurological improvement. By contrast, log *P* was not associated with the lipid-to-aqueous ratio or with early neurological outcome.

These in vivo findings support lipid-mediated xenobiotic redistribution after ILE and suggest that greater redistribution may be associated with early neurological improvement. This interpretation is consistent with current reviews, which regard the lipid sink or lipid shuttle as the main mechanism of ILE in most poisonings, while cardiometabolic effects appear to be most relevant in hemodynamically unstable LAST [19,20,39,40]. Partitioning has been observed in vitro when paired lipid and aqueous fractions were measured in human plasma or serum and xenobiotic concentrations were higher in the lipid fraction than in the aqueous [34,41,42]. In vivo support also exists. In swine given amiodarone, concentrations were higher in the lipid fraction and lower in the aqueous fraction after ILE, and this was associated with prevention of hypotension [36]. However, outside experimental models, most evidence linking redistribution to clinical outcome remains indirect [20].

If lipid capture and redistribution is an important mechanism, clinicians need a practical way to predict how much capture will occur for a given xenobiotic. Log *P* is an obvious first candidate because it is widely available and commonly used as a measure of lipophilicity in toxicology and drug design [30,31]. In this dataset, however, log *P* was not associated with the lipid-to-aqueous ratio or with short-term neurological outcome.

The limitations of log *P* in describing complex toxin partitioning are explicable. Log *P* is defined for the unionized form of a single compound in an idealized octanol-water system and does not account for pKa-dependent ionization [43,44]. In vivo, transfer into a transient intravascular lipid compartment depends not only on lipophilicity, but also on the balance between the neutral and ionized forms present at physiological pH [43,44].

This consideration may help explain why our findings differ from earlier studies. Many of those studies were done in vitro, examined only a single analyte, or focused on compounds such as local anesthetics or permethrin. These compounds are largely unionized at physiological pH and have very high log *P* values [21,29,33,45]. In those settings, log *P* may be enough to explain differences in partitioning. In the more complex setting of clinical patients and with other xenobiotics, however, in vitro lipophilicity may diverge markedly from real-world partitioning [19,44]. Current reviews therefore consider log *P* an incomplete predictor of in vivo partitioning after ILE and suggest that more physiologically relevant descriptors should also be considered [19,40,43]. Accordingly, caution is warranted against relying on log *P* alone for triage or efficacy prediction, despite its frequent use in clinical practice [4,32,45,46,47].

An alternative descriptor is the distribution coefficient at physiological pH 7.4 (log*D*7.4), which incorporates both lipophilicity and ionization state. Log*D*7.4 is therefore theoretically more relevant to in vivo behavior than log *P* [43,44,48]. However, log*D*7.4 is less widely available and may vary substantially according to the experimental method or computational model [25,43]. An in vitro study that evaluated both log *P* and log*D*7.4 found that log *P* correlated more strongly than log*D*7.4 with neurotoxic xenobiotic distribution behavior [29]. Therefore, although log*D*7.4 may be more physiologically relevant than log *P*, it still does not appear to provide a universally reliable predictor of xenobiotic partitioning or ILE efficacy across compounds. This is biologically plausible because in vivo partitioning is likely influenced by several additional determinants beyond lipophilicity and ionization state alone [19,40]. Xenobiotic capture by ILE does not necessarily occur only by dissolution into the lipid core. The phospholipid-coated droplet surface also carries a surface charge, so ionized parts of a xenobiotic may be trapped at the droplet surface through electrostatic interactions, whereas uncharged parts may move into the oily core and phospholipid layer [19]. Lipid sequestration and redistribution may therefore depend on more than singular partition-based descriptors.

Plasma protein binding may also influence sequestration, because a lower free fraction would be expected to reduce availability for lipid partitioning [19]. However, Barker et al. suggested that ILE may itself alter protein binding for some drugs, indicating that this relationship is more complex and unlikely to be simply linear [29].

Metabolites may add further complexity. In some intoxications, the clinically relevant toxic species may include both the parent compound and one or more metabolites, which may differ in lipophilicity, ionization state, and protein binding [19,29]. Similarly, concurrent intoxications and drugs used to treat the intoxication may also interact with ILE and may further complicate prediction of its therapeutic effect.

Volume of distribution may also help explain variation in the effect of ILE. It is a measure of how extensively a xenobiotic distributes out of plasma and into tissues. French et al. suggested that distribution properties, including volume of distribution, may help predict how strongly lipid emulsion reduces serum drug concentrations [49]. This is pharmacokinetically plausible, because xenobiotics with a large volume of distribution may have less access to the temporary intravascular lipid phase created by ILE.

The likely relevance of volume of distribution also emphasizes that the effect of ILE should not be viewed as a fixed endpoint, but as part of a continuous pharmacokinetic process. Xenobiotics may redistribute over time between plasma, tissues, and the intravascular lipid phase, while metabolism and excretion occur in parallel [19,20,40]. Experimental studies support this broader view, with changes reported not only in plasma fractions but also in target tissues such as the heart and brain [26,27,28,36]. Within this continuous pharmacokinetic process, it is notable that a higher lipid-to-aqueous ratio measured immediately after the end of ILE infusion was associated with early neurological improvement in our study. One possible explanation is that a substantial part of partitioning occurs soon after ILE administration. The finding that, by 4 h after completion of the infusion, the visible lipid phase was usually minimal or absent is consistent with this hypothesis and suggests that measurable intravascular partitioning into a distinct plasma lipid phase may be relatively short-lived.

In summary, these observations suggest that partitioning may indeed be relevant to ILE efficacy, but its contribution is unlikely to be fixed or fully captured by a single physicochemical descriptor, such as log *P*. In this context, recent attempts to develop more biologically informed, multivariable prediction models are especially relevant for further research [19,20,40,50].

Several important limitations of this study must be acknowledged. Although the initial sample size was substantial, post-exclusion numbers were modest, with very small toxin- and species-specific strata. Study precision and power were reduced, the risk of false-negative findings increased, and opportunities to adjust for clustering, explore dose–response relationships, or conduct reliable subgroup analyses were limited. Furthermore, the distribution of patients across toxin types and species was unequal, which limited generalizability and prevented robust statistical adjustment for clustering. This heterogeneity was further increased by variation in supportive treatments and by lack of control over the interval between exposure and presentation. These factors increase the risk of uncontrolled confounding and limit causal interpretation.

Multicenter, clinician-selected enrolment may have introduced selection bias through site-specific case mix and referral patterns, and the small post-exclusion sample prevented adjustment for site-level clustering. Furthermore, the population was likely biased towards severe intoxications because clinicians selected these cases for adjunctive therapy. Although this reflects real-world clinical practice, it may have increased between-patient variability and made true treatment effects harder to detect within the short 4–6 h observation window.

Protocol deviations in ILE administration were common and may have increased variability in the observed associations. The post hoc revised dosing criteria, i.e., a bolus followed by a continuous-rate infusion in the range totaling 15–30 mL/kg within 70 min, were consistent with published veterinary large multicenter data that report a wide range of doses including our range [51]. Although treatment was not fully standardized, the revised dosing range remained within published veterinary practice and may still have been sufficient to generate the intended intravascular lipid phase [19]. Once a circulating lipid phase exists, redistribution is thought to be primarily driven by major changes in physicochemistry rather than minor dose-related responses [19,32,40,41,50]. The broadened ILE dosing range nevertheless added variability between cases and reduced treatment uniformity within the final dataset. This should be considered when interpreting the associations observed in this study.

Concurrent supportive treatments also varied across cases. This reflected real-world hospital practice, but treatment heterogeneity may have masked or exaggerated the apparent effect of ILE.

Outcome was assessed within 4–6 h after treatment because this was the expected period during which the lipid sink would be effective [40]; however, to the authors’ knowledge, no validated approach for the timing of this assessment has been described. In most samples, the lipid fraction at 5 h after the start of ILE was very small. This suggests that the intravascular lipid phase, and any associated sequestration effect, had largely ended by that time. Earlier assessments might therefore have been more informative for capturing the early clinical effect of ILE. Also, later assessments might have been more informative for capturing the subsequent clinical course, including delayed recovery or redistribution. Such additional assessments were not feasible in the present multicenter clinical study.

Despite the broad GC–MS panel, many initially included cases were excluded because no xenobiotic was identified, suggesting diagnostic error, concentrations below the limit of detection, or that some cases were not true intoxications. In addition, a relatively high number of samples had concentrations below the limit of quantification and were imputed at half the limit of quantification for analysis. Because the aqueous-phase concentration is the denominator of the lipid-to-aqueous ratio, this may have inflated ratios when aqueous concentrations were very low. However, these values were confined to the lower end of the concentration range, and the analyses were performed on a log_10_ scale, which reduces the influence of extreme ratios arising from small denominators. Excluding all <LOQ observations was therefore not considered, as this would also have removed low-concentration cases without clearly improving representation of the underlying data.

In mixed exposures, the analyte with the highest measured concentration at T1 was used for the main analyses. This may not always have been the toxicologically most relevant agent, and it could have influenced clinical outcome, although cases with multiple detected xenobiotics were few. However, the primary aim was to study xenobiotic partitioning after ILE, and this approach reduced the impact of measurements near the limit of quantification, at which variability is larger.

Interpretation of the combined dog and cat analyses also requires caution. Cats and dogs are known to differ in drug metabolism and broader pharmacokinetic handling [52]. However, the primary mechanistic question in this study concerned partitioning into the transient intravascular lipid phase created by ILE, a process that is likely driven mainly by physicochemical interactions within plasma rather than by species-specific metabolic differences. We therefore considered the pooled analyses acceptable for an exploratory assessment of the overall direction of the association.

Neurological recovery scoring was structured and blinded but unvalidated. Dual independent scoring followed by consensus likely reduced random error but reintroduced subjectivity when initial agreement was suboptimal.

## 5. Conclusions

In clinical veterinary patients with suspected neurotoxicant exposure, ILE therapy sequestered xenobiotics into a plasma lipid phase in vivo, and higher sequestration was associated with early neurological improvement. These findings provide in vivo clinical support for lipid-mediated xenobiotic redistribution after ILE. However, because log *P* did not correlate with in vivo sequestration or early clinical outcome in this dataset, it should not be used as the sole predictor of xenobiotic plasma partitioning or the clinical efficacy of ILE therapy. Given the modest sample size, clinical heterogeneity, and limitations of outcome assessment, these findings should be interpreted as exploratory and hypothesis-generating. Future studies should consider other physicochemical and pharmacokinetic factors to help predict ILE efficacy in neurotoxicosis.

## Figures and Tables

**Table 1:** Xenobiotics detected in blood plasma following intravenous lipid emulsion therapy, frequency, and log *P* ^a^, in 27 dogs and 7 cats with suspected neurotoxicosis.

Xenobiotic	Dogs and Cats (n)	Dogs (n)	Cats (n)	Log *P*	CAS RN	PubChem CID
Permethrin	9	4	5	6.5	52645-53-1	40326
Δ9-Tetrahydrocannabinol	6	6	0	7.0	1972-08-3	16078
Diethyl phosphate	2	1	1	−0.2 ^b^	598-02-7	654
Alpha-chloralose	2	2	0	1.0	15879-93-3	7057995
Pyrilamine	2	2	0	3.3	91-84-9	4992
Caffeine	2	2	0	−0.1	58-08-2	2519
Menthone	1	1	0	3.0	89-80-5	26447
Baclofen	1	1	0	−1.0	1134-47-0	2284
Amitriptyline	1	1	0	4.9	50-48-6	2160
Metronidazole	1	1	0	0 ^b^	443-48-1	4173
Geraniol	1	1	0	2.9 ^b^	106-24-1	637566
Menthol	1	1	0	3.2	89-78-1	16666
2,5-Dimethoxy-p-cymene	1	1	0	3.4 ^b^	14753-08-3	6427071
Tramadol	1	1	0	2.6 ^b^	27203-92-5	33741
Olanzapine	1	1	0	3.0	132539-06-1	4585
3,6-Dimethoxy-9H-carbazole	1	1	0	2.8 ^b^	57103-01-2	644464
Pyriproxyfen	1	0	1	4.8 ^b^	95737-68-1	91753
Total	34	27	7	4.1 (−1.0 to 7.0) ^c^		

^a^ Log *P* = base-10 logarithm of the n-octanol/water partition coefficient; CAS RN: Chemical Abstracts Service Registry Number; PubChemCID: PubChem Compound Identifier (accessed April 2024, see also Data Availability Statement). ^b^ computed values (XLogP3 3.0) are shown when measured log *P* values were unavailable on PubChem. ^c^ Median (range).

**Table 2:** Spearman correlation between xenobiotic log *P* ^a^, plasma fraction concentration (lipid, aqueous), and the lipid-to-aqueous ratio ^b^ following intravenous lipid emulsion therapy in 34 companion animals with suspected neurotoxicosis.

	Ρ ^c^	*p* Value
Dogs (n = 27)		
Lipid	−0.24	0.22
Aqueous	−0.31	0.11
Lipid-to-aqueous ratio	0.14	0.48
Dogs + cats (N = 34)		
Lipid	−0.12	0.50
Aqueous	−0.26	0.14
Lipid-to-aqueous ratio	0.16	0.36

^a^ Log *P*: base-10 logarithm of the n-octanol/water partition coefficient (derived from PubChem; accessed April 2024). ^b^ lipid-to-aqueous ratio = lipid concentration divided by the aqueous concentration. ^c^ Spearman rank correlation coefficient.

**Table 3:** Strength of association xenobiotic log *P* ^a^ on xenobiotic plasma fraction concentration (lipid, aqueous), and the lipid-to-aqueous ratio following intravenous lipid emulsion therapy in 34 companion animals with suspected neurotoxicosis.

	GMR (95% CI) ^b^	*p* Value
Dogs (n = 27)		
Lipid	0.80 (0.60–1.07)	0.14
Aqueous	0.71 (0.54–0.94)	0.02
Lipid-to-aqueous ratio ^c^	1.13 (0.86–1.48)	0.39
Dogs + cats (N = 34)		
Lipid	0.91 (0.70–1.19)	0.50
Aqueous	0.78 (0.61–1.01)	0.07
Lipid-to-aqueous ratio	1.17 (0.92–1.48)	0.22

^a^ Log *P*: base-10 logarithm of the n-octanol/water partition coefficient (derived from PubChem; accessed April 2024) ^b^ GMR: back-transformed geometric mean ratio (10^*β*) on the original-scale outcome (mg/L) per +1 unit of log *P*. ^c^ lipid-to-aqueous ratio = lipid fraction concentration divided by the aqueous fraction concentration.

## Data Availability

De-identified participant-level data (species, site, ILE regimen, sampling timepoints, GC-MS concentrations, lipid-to-aqueous ratios, adverse events, and 4–6 h neurological outcome scores), the variable codebook, and the analysis code used to generate the results are available from the corresponding author upon reasonable request. Log *P* values were retrieved in April 2024 from PubChem; PubChem CIDs per xenobiotic are listed in Table 1.

## Supplementary Materials

The following supporting information can be downloaded at: https://www.mdpi.com/article/10.3390/ani16091352/s1, Table S1: Other treatments than intravenous lipid emulsion (ILE) therapy in 34 dogs and cats with suspected neurotoxicosis.; Table S2: Xenobiotic plasma concentrations (lipid fraction, aqueous fraction, and lipid-to-aqueous ratio) before (T0) and following (T1) intravenous lipid emulsion therapy in 27 dogs and 7 cats with suspected neurotoxicosis.
