# Supplementary material for: Effect of Intravenous Lipid Emulsion Therapy on Xenobiotic Plasma Partitioning and Short-Term Neurological Outcome in Companion Animals with Suspected Neurotoxicosis: A Case Series
Source: Animals (Basel). 2026 Apr 28;16(9):1352. doi: 10.3390/ani16091352 (PMC13163057; doi:10.3390/ani16091352)
Supplement: Supplementary file 1 [file animals-16-01352-s001.zip › animals-4179313-supplementary.pdf]

Supplementary Materials

Table S1. Other treatments than intravenous lipid emulsion (ILE) therapy in 34 dogs and cats with suspected neurotoxicosis. A single patient may have received more than one treatment and the same treatment at different time points.

Treatment	Before ILE[a]	During ILE[b]	After ILE[c]
sedatives/anti-convulsants	24	9	7
intravenous crystalloids	7	6	10
antiemetics	7	1	3
antibiotics	1	1	2
gastrointestinal decontamination	1	0	2
spasmolytics	1	0	1
gastric acid suppressants	0	0	1
immunomodulator	1	0	0

a After presentation but **prior to t**he start of the ILE infusion. b *W***hile** ILE was transfused (0–60 min after infusion started). c *First* administration recorded **>60 min after** ILE infusion had started.

Table S2. Xenobiotic plasma concentrations[a] (lipid fraction, aqueous fraction, and lipid-to-aqueous ratio[b]) before (T0) and following (T1) intravenous lipid emulsion therapy in 27 dogs and 7 cats with suspected neurotoxicosis.

Case	Xenobiotic	T0 (mg/L)	T1 Lipid (mg/L)	T1 Aqueous (mg/L)	T1 Lipid-To-Aqueous Ratio
Dogs					
1	2,5-Dimethoxy-p-cymene	0.23	0.44	0.22	2.00
2	Δ9-Tetrahydrocannabinol	2.11	1.30	0.82	1.59
3	Δ9-Tetrahydrocannabinol	0.01	0.61	0.50	1.22
4	Δ9-Tetrahydrocannabinol	0.03	0.04	<0.01	8.00
5	Δ9-Tetrahydrocannabinol	0.02	0.41	<0.01	82.00
6	Δ9-Tetrahydrocannabinol	0.02	0.03	<0.01	6.00
7	Δ9-Tetrahydrocannabinol	<0.01	0.01	<0.01	2.00
8	Alpha-chloralose	<0.01	0.07	0.12	0.58
9	Alpha-chloralose	<0.01	<0.01	<0.01	1.00
10	Amitriptyline	0.01	0.11	0.05	2.20
11	Baclofen	5.90	6.50	2.40	2.71
12	Caffeine	0.50	0.40	0.30	1.33
13	Caffeine	0.10	0.21	<0.01	4.20
14	Diethyl phosphate	<0.01	3.69	<0.01	73.8
15	3,6-Dimethoxy-9H-carbazole	1.51	4.89	<0.01	978
16	Geraniol	0.57	0.71	0.55	1.29
17	Menthanon	0.84	0.48	<0.01	9.60
18	Menthol	<0.01	4.17	1.69	2.47
19	Metronidazole	2.63	0.23	6.20	0.04
20	Olanzapine	<0.01	0.13	0.16	0.81
21	Permethrin	<0.01	0.02	0.01	2.00
22	Permethrin	0.20	6.45	0.38	16.97
23	Permethrin	<0.01	0.01	<0.01	2.00
24	Permethrin	0.01	0.14	0.01	14.00
25	Pyrilamine	0.52	6.09	0.23	26.48
26	Pyrilamine	<0.01	2.06	0.17	12.12
27	Tramadol	0.10	0.45	0.10	4.50
Cats					

1	Diethyl phosphate	<0.01	0.34	<0.01	6.80
2	Permethrin	IPV[c]	1.07	0.01	107.00
3	Permethrin	0.46	15.65	1.38	11.34
4	Permethrin	0.22	9	0.72	12.50
5	Permethrin	0.08	0.47	0.16	2.94
6	Permethrin	0.21	0.23	0.04	5.75
7	Pyriproxyfen	<0.01	1.5	<0.01	300

a “<” indicates a concentration below the level of quantification (LOQ 0.01 mg/L). *b* lipid-to-aqueous ratio = lipid fraction concentration divided by the aqueous fraction concentration. *c* IPV = insufficient plasma volume for analysis.
